# Phenolic Content and Antioxidant, Antihyperlipidemic, and Antidiabetogenic Effects of *Opuntia dillenii* Seed Oil

**DOI:** 10.1155/2020/5717052

**Published:** 2020-10-06

**Authors:** Mohamed Bouhrim, Nour Elhouda Daoudi, Hayat Ouassou, Amina Benoutman, El Hassania Loukili, Abderrahim Ziyyat, Hassane Mekhfi, Abdelkhaleq Legssyer, Mohammed Aziz, Mohamed Bnouham

**Affiliations:** ^1^Laboratory of Bioresources, Biotechnologies, Ethnopharmacology, and Health, Faculty of Sciences, University Mohamed First, Oujda, Morocco; ^2^Laboratory of Biology, Environment, and Sustainable Development, Higher Normal School, Abdelmalek Essaadi University, Tetouan, Morocco; ^3^Laboratory of Applied Analytical Chemistry, Materials and Environment, Department of Chemistry, Faculty of Sciences, Mohammed First University, Oujda, Morocco

## Abstract

*Opuntia dillenii* (Ker-Gawl.) Haw. is a medicinal plant that is widely used by the Moroccan population to treat many diseases, thanks to its richness in bioactive molecules. This study aims to evaluate the total phenolic content and antioxidant, antihyperlipidemic, and antidiabetogenic activities of *O. dillenii* seeds oil (ODSO), in vivo. The 2,2-diphenyl-1-picrylhydrazyl (DPPH) scavenging assay and the Folin–Ciocalteu method were applied in this study to determine antioxidant activity and total phenolic content of ODSO, respectively. The antihyperlipidemic effect of the ODSO (2 ml/kg) was evaluated in the high-fat diet-fed albino mice, relying on lipid profile, blood glucose, and growth performance variations. Moreover, the preventive effect of ODSO was evaluated against alloxan monohydrate-induced diabetes in albino mice. ODSO had the highest total phenolic content (518.18 ± 14.36 mg EAC/kg) and DPPH scavenging activity (IC_50_ = 0.38 ± 0.08 mg/mL). Furthermore, ODSO showed a significant antidiabetogenic effect by reducing bodyweight loss, blood sugar level rise, and mortality rate caused by alloxan in albino mice. Then, ODSO has exhibited a significant antihyperlipidemic effect by improving the lipid profile disorder and glucose level rise in the blood, produced by the high-fat diet-fed albino mice. Results suggest that antidiabetogenic and antihyperlipidemic activities of ODSO correlate to the phenolic content and antioxidant activity of this oil. Hence, this plant could be a significant source of medically important critical compounds.

## 1. Introduction

The cactus family consists of about 1,500 species belonging to the genus *Opuntia*, distributed in Africa, the Mediterranean countries, Northern Mexico, the Southwestern United States, and other regions [1]. *O. dillenii* is a species of cactus, and it is found in the northeast and west Morocco. Its fruit is consumed exclusively in the form of fresh fruit. The fruit of *O. dillenii* is used by the population in traditional medicine to treat diabetes, inflammation, analgesic, and gastric ulcers [2]. Much information about the cladode, juice, and pear of *O. dillenii* is available but little about the nutritional value and biological activities of its seed oil. Ghazi et al. investigated the *O. dillenii* seed oil composition and its chemical characteristics. They showed that this product belongs to the kind of oils that have increased degrees of unsaturated fatty acids, in which linoleic acid is the highest fatty acid, and *ß*-sitosterol is the majority sterol. At the same time, *γ*-tocopherol is the only vitamin *E* present in this oil [3]. Besides, it is characterized by the presence of other components such as phenols [4]. All of which may contribute to human health [5]. These antioxidant components have pharmacological properties that can prevent and reduce oxidative stress-related diseases [6]. As a result, interest has recently increased in the use of new natural antioxidants, mainly of plant origin. Several studies that have been performed on the fruit seeds oil of this plant have shown that this oil has significant antioxidant activity [6, 7], the hepatoprotective effect [9], and the curative antidiabetic effect [10]. Currently, no study has been carried out on the preventive effect of this oil against the onset of diabetes or its antihyperlipidemic effect in high-fat diet-fed mice. For that, the objective of this study is to evaluate the antidiabetogenic effect of ODSO from Morocco against alloxan-induced diabetic mice and searching for the mechanism of this effect by or through the analysis of their total phenolic content and antioxidant activity. Also, the antihyperlipidemic effect in high-fat diet-fed mice was evaluated.

## 2. Materials and Methods

### 2.1. Chemicals

DPPH and ascorbic acid were purchased from Sigma-Aldrich (USA). Alloxan monohydrate (Allx monohydrate 98%, ACROS Organics), Folin–Ciocalteu, and glucose oxidase/peroxidase reagent glucose kit were purchased from Sigma-Aldrich (Steinheim, Germany). All other reagents and chemicals used are of analytical grade.

### 2.2. Collection of Plant Material

The fresh fruits of *O. dillenii* from the Essaouira region of Morocco were used in this study. These fruits were harvested in February 2018 and identified by a botanical expert Mohammed Fennan, from the Scientific Institute of Mohammed V University. The specimen was deposited at Mohammed First University, Oujda, Morocco, under the reference number HUMPOM 351.

### *2.3. Opuntia dillenii* Seeds Preparation

The fruits of *O. dillenii* were peeled, the seeds were separated and cleaned with distilled water, and then, the seeds were dried and crushed in a blender until they were fine and in the form of a homogeneous powder; this powder was stored at −20°C until use.

### 2.4. Oil Extraction

To extract the oil from the seeds, 500 mL of the petroleum ether was added to 100 g of the seeds powder, and the mixture was stirred at room temperature for 24 hours. After filtration, a rotary evaporator was used to remove the solvent at a temperature of 40°C; the oil was dried and stored at 4°C.

### 2.5. Phenolic Extraction

The extraction of the total phenol compounds of ODSO was carried out by adding 1 mL of methanol 80% to 1 g of ODSO. Subsequently, the mixture was vortexed for 10 min; the whole is centrifuged for 15 min at 3800 rpm and room temperature. The hydroalcoholic extract of ODSO (supernatant) was recovered in a 5 mL volumetric flask, and the volume of the extract was adjusted to 5 mL with methanol 80%.

### 2.6. Determination of Total Phenolic Contents

The determination of the total phenols of ODSO was carried out according to the Folin–Ciocalteu method [11]. Indeed, the reaction mixture contains 0.5 mL of the hydroalcoholic extract of ODSO, 0.5 mL of MethOH 80%, 5 mL of Na_2_CO_3_ 10%, and 1 mL of the Folin–Ciocalteu solution. The mixture was vortexed and incubated in the dark at room temperature for 60 minutes. Thereafter, the absorbance was measured at 750 nm by a spectrophotometer. The values are expressed in mg equivalent of caffeic acid.

### 2.7. Diphenyl-1-Picrylhydrazyl Radical Scavenging Assay

The DPPH scavenging assay was performed according to the method described by Liu [12], with some modifications. The reaction mixture consists of 1.5 mL of the ethanolic solution of ODSO in a concentration series (6, 4, 1, 0.40, and 0.10 mg/mL) and 1 mL of ethanolic solution of DPPH (0.001%). The mixture was shaken by vortex, and after 30 min of incubation, the absorbance was immediately determined by a spectrophotometer at 517 nm. Ascorbic acid, a standard antioxidant, was used as a reference. All assays were performed in triplicates. The scavenging activity of the samples was calculated according to the following formula:

(1) $$\begin{array}{c} \text{DPPH scavenging percentage }\left(\text{\%}\right)=100\times \left[\frac{\left(\text{Ac}-\text{As}\right)}{\text{Ac}}\right], \end{array}$$

where Ac and As is the absorbance of the control and tested samples, respectively, at the measurement time.

### 2.8. Animals and Housing

Swiss albino mice (19 weeks old) have been used in this study. They have been housed in Makrolon cages under standard laboratory conditions (12 h light/12 h darkness, 21 ± 2°C). The animals have been given standard pellets diet and water ad libitum throughout the experimental period. All animals have been cared for in compliance with the internationally accepted guide for the care and use of laboratory animals, published by the US National Institutes of Health (NIH Publication No. 85-23, Revised in 1985).

### 2.9. Effect of *Opuntia dillenii* Seed Oil on Allx-Induced Diabetes

#### 2.9.1. Diabetes Induction

The onset of diabetes in mice was induced by an intraperitoneal injection of a single dose of alloxan monohydrate (100 mg/kg) [13]. The diabetogenic agent was freshly prepared in a phosphate-citrate buffer (pH = 4.5) and injected into fasting mice for 14 hours.

#### 2.9.2. Experimental Protocol Design

To evaluate the effect of ODSO treatment on the incidence of Allx-induced diabetes, the mice were randomly separated into five groups: control group, Allx group, Allx + ODSO (1 mL/kg), Allx + ODSO (2 mL/kg) treated groups, and Allx + Asco (2 mg/kg) group. During three days, treated animals received orally the ODSO (1 and 2 mL/kg) and ascorbic acid (2 mg/kg) to their respective groups, followed by a single dose injection of Allx at 1 h of the time interval. The treatment was sustained for a week after the Allx administration. Control and Allx groups received oral administration of distilled water (10 mL/kg) followed by an intraperitoneal injection of phosphate-citrate buffer and Allx, respectively. Blood was withdrawn from the mice tail vein, from overnight fasting mice, and glycemia was measured at the start and the end of the study, using a diagnostic kit. Bodyweight was determined before and after the treatment period [13].

### 2.10. Antihyperlipidemic Effect of *Opuntia dillenii* Seed Oil on Mice Fed a High-Fat Diet

#### 2.10.1. Preparation of the High-Fat Diet

The high-fat diet was prepared daily according to the method described by Harnafi et al. [14]; this diet consisted of the regular chow diet (Society SONABETAIL, Oujda, Morocco), cholesterol 2%, fat 16%, and deoxycholic acid 0.2%.

#### 2.10.2. Experimental Protocol Design

The albino mice were divided into five groups (*n* = 6; ♂/♀ = 1). NCG represented the normolipidemic control group, and the mice received distilled water (10 mL/kg). HCG represented the hyperlipidemic control group, and the mice received freely the high-fat diet and daily received distilled water (10 mL/kg). Moreover, OCG represented the ODSO group, and the mice received freely the high-fat diet and were daily treated by the ODSO at a dose of 2 mL/kg.

After the end of the treating period (30 days), the mice were fasted for 14 hours and lightly anesthetized with diethyl ether. The blood samples were then taken from their retroorbital sinus into heparinized tubes. The blood samples were immediately centrifuged (2500 rpm/15 min), and plasma was used for lipid analysis.

#### 2.10.3. Biochemical Analysis

The biochemical parameters have been measured in plasma. Blood glucose, total cholesterol (TC), triglycerides (TG), high-density lipoprotein cholesterol (HDL-c) levels were measured by the commercial kits. All tests were performed with the COBAS INTEGRA® 400-Plus analyzer.

The atherogenic index was calculated by the following formula:  AI=(Total cholesterol) − (HDL − C)/HDL − C, and in the LDL_C/HDL_C, the ratio was calculated as the ratio of plasma LDL-C to HDL-C levels.

### 2.11. Statistical Analysis

The results obtained have been analyzed by Graph Pad Prism 5 and been expressed as mean ± standard error of the mean (SEM). The results have been analyzed by one-way ANOVA. The difference was considered statistically significant when *P* < 0.05.

## 3. Results and Discussion

### 3.1. Total Phenolic Content

The determination of total phenols by the Folin–Ciocalteu method revealed that the average content of ODSO extracted by the ether petroleum in phenols equivalent milligrams of caffeic acid per 1000 g of oil is equal to 518.18 ± 14.36 mg/1000 g oil. In a study conducted by Koubaa et al., ODSO from the Sfax region (Tunisia) extracted using SC-CO2 was more enriched in polyphenols (172.2 ± 11.9 *μ*g GAE/g of oil) than that extracted with hexane (76.0 ± 6.9 *μ*g GAE/g of oil). Moreover, the polyphenol profiles showed that the SC-CO2 process gave a higher yield of compounds (45) compared with the extraction with hexane (11). In general, among the most abundant polyphenols in this oil, we find catechol, cinnamic acid, 3-phenylpropionic acid, psoralen, syringic acid, sinapaldehyde, 3′-O-methylcatechin, (+)-gallocatechin, bisdemethoxycurcumin, 4′-O-methyl-(-)-epicatechin 3′-O-glucuronide, and viscutin 1 [8]. However, phenolic compounds and flavonoids are known by a high antioxidant effect against various reactive oxygen and nitrogen species [15].

### 3.2. DPPH Scavenging Assay

**Figure 1:**
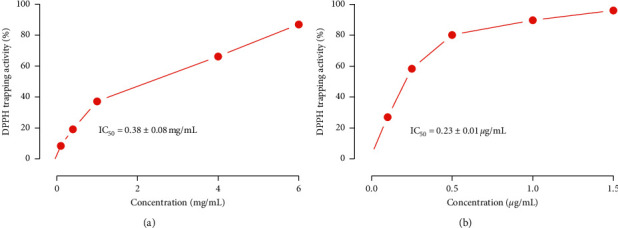
Effect on DPPH scavenging by ODSO (a) at various concentrations. Ascorbic acid (b) was used as a standard antioxidant, (*n* = 3); ODSO, *O. dillenii* seeds oil.

The antioxidant effect of ODSO was evaluated over a range of concentrations, and the results of the DPPH scavenging effect are shown in Figure 1. ODSO exhibited significant antioxidant activity with an IC_50_ value of 0.38 ± 0.08 mg/mL. Moreover, the IC_50_ of ascorbic acid was 0.23 ± 0.01 *μ*g/mL, and it is lower than that of ODSO. Additionally, the antioxidant ability of ODSO increased proportionally to its concentration in the mixture. Moreover, several studies evaluating this oil have shown that it has significant antioxidant activity [7, 12, 16]. However, antioxidant activity of ODSO was also concentration-dependent [12]. Besides, in another study, antioxidant activity of ODSO was assessed using a DPPH scavenging assay. The results showed that antioxidant activity of ODSO was 27.21 ± 0.075 *μ*L/mL and is higher than that of ascorbic acid (IC_50_ = 16.56 ± 0.019 *μ*g/mL). Besides, antioxidant activity of this oil was proved to be concentration-dependent [7]. This essential antioxidant activity is an indicator of the presence of antioxidant molecules in this oil, such as phenolic compounds. Antioxidants are substances that protect our body from damage caused by free radicals by removing free radicals and preventing their oxidation [6].

### 3.3. Effect of ODSO Administration on Alloxan Diabetes Induction

#### 3.3.1. Effect on Survival Rate

**Figure 2:**
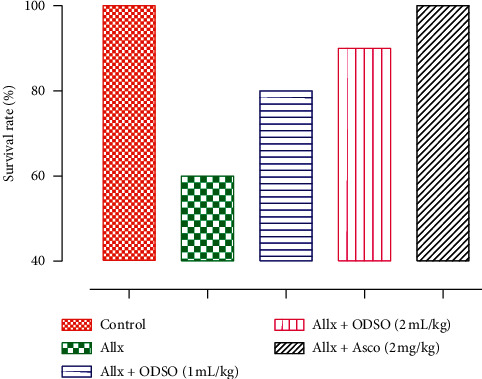
The effect of ODSO and ascorbic acid on the survival rate of Swiss albino mice after Allx administration. *n* = 10; ODSO, *O. dillenii* seeds oil; Allx, alloxan.

The treatment of the mice with ODSO at doses of 1 and 2 mL/kg enhanced the survival rate 80% and 90%, respectively, after Allx injection (100 mg/kg), compared to the group treated only with Allx 60%. Several experimental studies have shown that Allx induces a sudden increase in insulin release immediately after injection [17]. Moreover, Allx induced death of mice due to fatal hypoglycemic seizures [18]. In the same way, ascorbic acid administration at the dose 2 mg/kg prevented mortality due to the Allx administration compared to the Allx group 100% (Figure 2). These results suggest that the ODSO treatment significantly attenuated the development of the diabetes mellitus induced by the Allx in Swiss albino mice. Moreover, these results are consistent with those found by Ali et al. [13]. The ODSO is rich in phenolic compounds that can extinguish free radicals and therefore improve survival in mice treated with ODSO and Allx [13]. Also, ODSO is rich in linoleic acid [3], and this polyunsaturated fatty acid has prevented 100% mortality of animals caused by Allx [19].

#### 3.3.2. Effect on Blood Glucose Level

**Figure 3:**
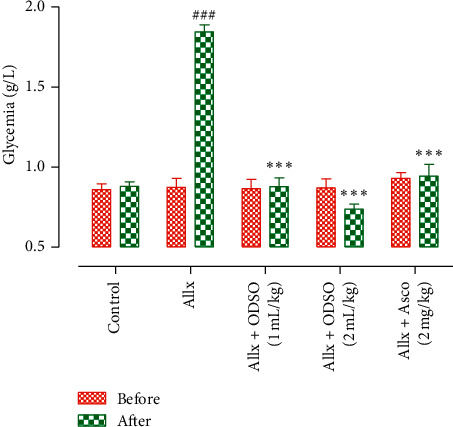
Effect of oral administration of the ODSO and the ascorbic acid on blood glucose level before (day 1) and after (day 7) Allx intraperitoneal injection in mice. The values are presented in mean ± SEM. *n* = 6-10, ^###^*P* < 0.001 compared to the control group and ^*∗∗∗*^*P* < 0.001 compared to the Allx control group.

The effect of ODSO intake on the glycemic level of Allx-treated mice is shown in Figure 3. The injection of Allx induced a significant (*P* < 0.001) increase in the fasting blood glucose of the diabetic control group mice compared to the normal control group mice. The administration of the ODSO at two doses 1 and 2 mL/kg with Allx in mice significantly (*P* < 0.001) decreased the incidence of Allx-provoked hyperglycemia in the ODSO-treated group in comparison with the group treated only with Allx. The treatment with ascorbic acid at 2 mg/kg was attenuated significantly (*P* < 0.001), and the alloxan provoked hyperglycemia compared to the group treated only by Allx. These results suggest that the ODSO treatment significantly attenuated the development of the diabetes mellitus induced by Allx in Swiss albino mice. Moreover, these results are consistent with those found by Ali et al. [13]. Indeed, Allx is known as a chemical molecule that is used in the laboratory to produce diabetic animal models [20]. It is an unstable chemical compound that causes necrosis of pancreatic islet beta cells [21]. This effect is explained by the fact that the presence of Allx and its reducing products in the *ß*-cells of the organism leading to the production of superoxide radicals (O2•) [22]. These superoxide radicals undergoing dismutation eventually lead to the formation of hydrogen peroxide (H_2_O_2_) and react with ferrous (Fe^2+^) to produce hydroxyl radicals (OH•), a highly oxidizing agent [23]. This imbalance in the redox balance causes necrosis and death of pancreatic beta cells [21]. Therefore, permanent hyperglycemia, the diabetogenic effect of the chemical agent, which is Allx, passes through the oxidation process by the production of free radicals [21]. High levels of free radicals are implicated in the development of many chronic diseases, according to scientific research [24]. In type 1 diabetes, free radicals also participate in the autoimmune reaction, leading to the destruction of pancreatic cells and the alteration of insulin synthesis and secretion [25]. Therefore, agents with antioxidant properties may have the potential for limiting diabetes progression [26]. This effect could be mainly due to the protection of Langerhans islets against the toxicity of free radicals produced by Allx. It is suggested that the ODSO contains components that have antioxidant properties and maintain the balance of the antioxidant/prooxidant balance of the *ß*-pancreatic cells. In this study, the ODSO showed important antioxidant activity. It could be that this antidiabetogenic effect goes through its antioxidant effect. Indeed, the chemical analysis of this ODSO showed that it contains a huge quantity of unsaturated fatty acids, wherein linoleic acid is the majority of polyunsaturated fatty acids. It has been confirmed that oil rich in linoleic acid prevents Allx-provoked diabetes in rats by preserving redox homeostasis in *ß*-cells and increasing the amount of cytoprotective antioxidant compounds such as glutathione reductase, superoxide dismutase, and vitamin E [27]. Moreover, the oil of *Opuntia ficus-indica* was shown to have an antidiabetogenic effect [13], and this oil was found rich in linoleic acid [28, 29]. Moreover, the ODSO is rich in a metabolite of linoleic acid, *γ*-linolenic, and pretreatment with *γ*-linolenic acid was shown to prevent 100% of the incidence of Allx-induced diabetes in rats [30]. Besides, the ODSO was found in this study that is rich in *γ*-tocopherol. *γ*-Tocopherol has been reported to have anti-inflammatory and antioxidant activities [31]. Tomasch et al. who demonstrated that administration of the oil rich in *γ*-tocopherol improves the antioxidant plasma capacity in healthy male volunteers have confirmed these results [32].

#### 3.3.3. Effect on Bodyweight

The bodyweight variation of mice is shown in Table 1. Allx caused a significant decrease in bodyweight in the Allx group (*P* < 0.001) compared to the control group, which asserts the corruption of necessary proteins due to diabetes [33]. However, the ODSO intake with Allx inhibited significantly (*P* < 0.001) the bodyweight loss in treated groups compared to the group treated only with Allx. These results suggest that the ODSO treatment significantly attenuated the development of the diabetes mellitus induced by Allx in Swiss albino mice. Moreover, these results are consistent with those found by Ali et al. [13].

### 3.4. Effect of the ODSO on High-Fat Diet-Induced Lipid Metabolism Disturbance in Mice

#### 3.4.1. Bodyweight and Relative Weight of Organs

Bodyweight gain, relative kidney weight, and relative liver weight were not significantly different between all groups treated (Table 2). The high-fat-diet-fed mice produced did not cause significant increases in bodyweight gain, relative kidney weight, and relative liver weight compared with the standard diet-fed mice. The daily intake of ODSO of the high-fat diet-fed in mice attenuated the increased bodyweight, relative kidney weight, and relative liver weight of high-fat diet-fed mice and did not induce a significant difference compared to the high-fat diet-fed mice. These results are in good agreement with the results found by Kim et al. It was found that the perilla oil provoked an increase in the bodyweight gain in the high-fat-diet-fed mice, compared to the high-fat diet mice, but this increase was not significant [34].

#### 3.4.2. Lipid Parameters

Table 3 presents the effect of the daily intake of the ODSO on lipid parameters on a high-fat diet-induced lipid metabolism disturbance in mice. The administration of the high-fat diet in mice has induced a significant increase in the cholesterol, HDL cholesterol/total cholesterol (%), triglycerides, and the atherogenic index levels, but it did not significantly decrease in serum levels of the HDL cholesterol (g/L) level compared to the normal control group. However, the daily intake of the ODSO has significantly attuned the increase in the cholesterol, HDL cholesterol/total cholesterol (%), triglycerides, and the atherogenic index levels and did not affect the HDL cholesterol (g/L) level compared to the high-fat-diet group. Until now, there are no data about the antihyperlipidemic effect of the ODSO in the high-fat-diet-fed animals. In other studies, the antihypercholesterolemic effect of other oils was evaluated. However, a study conducted by Kim et al. proved that the perilla oil or pomace olive oil intake significantly attenuated the increase in the plasma cholesterol in the high-fat diet-fed mice, and these results are in agreement with our findings [34, 35]. Also, they showed that perilla oil treatment reduced the hepatic triglyceride levels compared with the high-fat diet-fed mice [36]. According to the research carried out on the oil of another *Opuntia* species from Tunisia, the supplementation of rat feed by 25 g/kg seed oil has induced a decrease of blood cholesterol and low-density lipoprotein (LDL); in contrast, high-density lipoprotein (HDL) remained almost unchanged during the treatment [37]. In another study conducted by Ennouri et al., it was shown that dietary supplementation of *O. ficus-indica* seed oil at a dose of 25 g/kg could be useful in reducing the atherogenic risk factors in the rat. The enrichment of the diet by this oil has induced a very pronounced lipid-lowering effect in the treated rats compared to the rats receiving a control diet. Indeed, a significant reduction in total cholesterol and the ratio of HDL cholesterol to total cholesterol was significantly higher than that of the control group. Also, the atherogenic index was significantly lower in rats treated with an oil-rich diet than that in controls [38]. The chemical analysis of the ODSO was shown that this oil is rich in polyunsaturated fatty acid (linoleic acid) [3]. The most important function of polyunsaturated fatty acids is the provision of a unique class of precursors for conversion to metabolites, which regulate the lipid profile [39]. Moreover, mammals use linoleic acid as a precursor to eicosapentaenoic acid (EPA) and docosahexaenoic acid (DHA) that are important for lipid metabolic processes [40]. Therefore, Kim et al. suggest that perilla oil may affect the expression of lipid metabolic proteins and shows that perilla oil increased *p*-AMPK, *p*-ACC, and lipolytic proteins but decreased lipogenic proteins [34]. It is universally accepted that the consumption of polyunsaturated vegetable oils will reduce plasma cholesterol levels. Also, the effect of polyunsaturated fatty acids on plasma triglyceride levels has been shown in several studies. And HDL cholesterol levels have been affected variable by linoleic acid-rich diets [39].

#### 3.4.3. The Glucose Level

**Figure 4:**
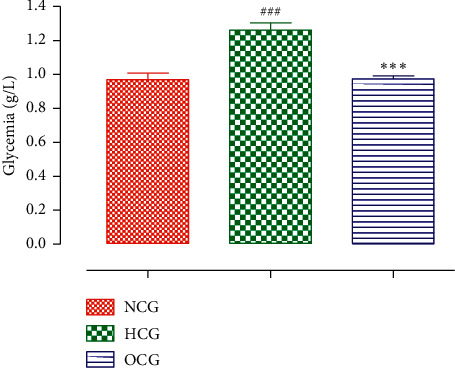
Effect of the ODSO on the glucose level. The values are presented in mean ± SEM. *n* = 6–10; ^###^*P* < 0.001 compared to the control group; ^*∗∗∗*^*P* < 0.001 compared to the Allx control group.

The result in Figure 4 shows the effect of ODSO daily intake on the glucose level in the high-fat diet-fed mice. The high-fat diet-fed mice have produced a significant increase in plasma glucose compared to the standard diet-fed mice. The abnormal lipid metabolism produced by the high-fat diet-fed mice is known to cause insulin resistance disease [36]. The daily intake of ODSO to the high-fat diet-fed mice has significantly attenuated the increase in the plasma glucose compared to the high-fat diet-fed mice. A study conducted by Bouhrim et al. proved that ODSO has a hypoglycemic effect in diabetic rats [10]. Ali et al. found similar results. It was found that cactus pier seeds oil, a natural product rich in unsaturated fatty acid [28, 29], has antihyperglycemic property in diabetic rats [41]. Moreover, in the majority of studies on vegetable oils, it seems that they increase the tolerance to glucose in the different animal models following treatment in repeated doses, and this is by the regeneration of beta cells or the decrease of resistance to insulin [42]. Other studies have also shown an antihyperglycemic effect of different types of fixed oils (pomace olive oil) in the high-fat diet-fed mice [35]. According to a research conducted on *O. ficus-indica* grown in Tunisia, the addition of *O. ficus-indica* seed oil in the diet of rats at a dose of 25 g/kg has shown a significant hypoglycemic effect. Indeed, the enrichment of the diet with the oil induced a significant decrease in blood glucose (22%) and a significant increase of glycogen concentration in the liver and muscle [37]. In a study using L6 muscle cells, polyunsaturated fatty acids improved glucose capture after insulin resistance caused by palmitic acid. Also Park et al. reported that essential fatty acids (linoleic and alpha-linolenic) improve glucose capture by muscle C2C12 cells-rendered insulin resistance by palmitic acid [43]. In a study, in which 3T3-L1 adipocyte lines were used, Prabhakar and Doble demonstrated that vanillic acid, one of the phenolic compounds of ODSO, increases glucose capture by treated adipocytes by approximately three times by inducing the PI3K pathway leading to increased translocation of GLUT4 to the cell surface [44].

## 4. Conclusion

In conclusion, the ODSO was effective in preventing diabetes in mice caused by the alloxan monohydrate. This effect could be due to their phenolic compounds, which could be reacted alone, or in synergy to scavenge the free radicals produced by the alloxan. Moreover, the capacity of their unsaturated fatty acids, sterols, and vitamin *E* to improve antioxidant activity in pancreatic beta cells. Consequently, further studies need to be carried out to find out the mechanism of action of these bioactive compounds present in this oil to prevent the onset of diabetes. Moreover, ODSO has shown a significant antihyperlipidemic effect in mice. Hence, this effect could be linked to its richness in polyunsaturated fatty acids, phytosterols, vitamin *E*, and phenolic compounds.

## Figures and Tables

**Table 1:** Changes in the body weight before and after Allx intraperitoneal administration in the ODSO and the ascorbic acid-treated mice groups.

Groups	Bodyweight (g)		Bodyweight gain (g)
	Initial	Final	
Control	28.75 ± 0.60	29.91 ± 0.49	01.16 ± 0.17
Allx	28.50 ± 0.58	27.70 ± 0.56	−00.80 ± 0.18^###^
Allx + ODSO (1 mL/kg)	25.80 ± 0.63	25.81 ± 0.59	00.01 ± 0.17^*∗∗∗*^
Allx + ODSO (2 mL/kg)	26.06 ± 0.80	26.94 ± 0.59	00.88 ± 0.47^*∗∗∗*^
Allx + Asco (2 mg/kg)	28.15 ± 0.48	28.80 ± 0.34	00.65 ± 0.22^*∗∗∗*^

The values are presented in mean ± SEM. *n* = 6–10; ^###^*P* < 0.001 compared to the control group; ^*∗∗∗*^*P* < 0.001 compared to the Allx control group.

**Table 2:** Effect of the ODSO on growth performance.

Parameters	NCG	HCG	OCG
Initial weight (g)	22.34 ± 0.52	23.37 ± 0.82	22.48 ± 0.29
Average weight gain (g/rat)	5.59 ± 0.81	7.42 ± 0.21	5.29 ± 1.91

The relative weight of organs			
Kidney (g/100 g BW)	0.62 ± 0.18	0.72 ± 0.12	0.78 ± 0.07
Liver (g/100 g BW)	3.91 ± 0.81	4.61 ± 0.72	4.16 ± 0.72

**Table 3:** Effect of the ODSO on the lipids parameters levels.

Parameters	NCG	HCG	OCG
Cholesterol (g/L)	0.54 ± 0.08	0.72 ± 0.04^###^	0.61 ± 0.04^*∗*^
HDL cholesterol (g/L)	0.41 ± 0.05	0.36 ± 0.03	0.43 ± 0.06
HDL cholesterol/total cholesterol (%)	0.77 ± 0.14	0.49 ± 0.05^##^	0.71 ± 0.11^*∗*^
Triglycerides (g/L)	0.43 ± 0.03	0.69 ± 0.09^###^	0.51 ± 0.09^*∗∗*^
Atherogenic index	0.33 ± 0.24	1.03 ± 0.22^###^	0.45 ± 0.22^*∗∗*^

The values are presented in mean ± SEM. *n* = 6–10; ^###^*P* < 0.001 and ^##^*P* < 0.01 compared to NCG; ^*∗∗*^*P* < 0.01 and ^*∗*^*P* < 0.05 compared to HCG.

## Data Availability

The data used to support the findings of this study are available from the corresponding author upon request.
